# Transcriptomic Analysis of the Ion Channelome of Human Platelets and Megakaryocytic Cell Lines

**DOI:** 10.1160/TH15-11-0891

**Published:** 2016-06-09

**Authors:** Joy R. Wright, Stefan Amisten, Alison H. Goodall, Martyn P. Mahaut-Smith

**Affiliations:** 1 Department of Molecular and Cell Biology, University of Leicester, Leicester, UK; 2 Division of Diabetes and Nutritional Sciences, Kings College, London, UK; 3 Department of Cardiovascular Sciences, University of Leicester and NIHR Cardiovascular Biomedical Research Unit, Glenfield Hospital, Leicester, UK

**Keywords:** Platelet physiology, megakaryocytes, molecular biology methods, receptors

## Abstract

Ion channels have crucial roles in all cell types and represent important therapeutic targets. Approximately 20 ion channels have been reported in human platelets; however, no systematic study has been undertaken to define the platelet channelome. These membrane proteins need only be expressed at low copy number to influence function and may not be detected using proteomic or transcriptomic microarray approaches. In our recent work, quantitative real-time PCR (qPCR) provided key evidence that Kv1.3 is responsible for the voltage-dependent K^+^ conductance of platelets and megakaryocytes. The present study has expanded this approach to assess relative expression of 402 ion channels and channel regulatory genes in human platelets and three megakaryoblastic/erythroleukaemic cell lines. mRNA levels in platelets are low compared to other blood cells, therefore an improved method of isolating platelets was developed. This used a cocktail of inhibitors to prevent formation of leukocyte-platelet aggregates, and a combination of positive and negative immunomagnetic cell separation, followed by rapid extraction of mRNA. Expression of 34 channel-related transcripts was quantified in platelets, including 24 with unknown roles in platelet function, but that were detected at levels comparable to ion channels with established roles in haemostasis or thrombosis. Trace expression of a further 50 ion channel genes was also detected. More extensive channelomes were detected in MEG-01, CHRF-288–11 and HEL cells (195, 185 and 197 transcripts, respectively), but lacked several channels observed in the platelet. These “channelome” datasets provide an important resource for further studies of ion channel function in the platelet and megakaryocyte.

## Introduction

Platelets play an essential role in haemostasis and thrombosis and contribute to immune responses, angiogenesis, lymph vessel development and liver regeneration (1). Platelets also have a pathophysiologic role in cancer progression and development of atherosclerosis. Ion channels are transmembrane proteins with ubiquitous roles in homeostasis and cellular activation. Approximately 20 ion channels have been previously reported in platelets or their nucleated precursor cell, the megakaryocyte (2). Whereas some channels, such as ATP-gated P2X1 channels and store-operated Orai1 channels, have defined roles in platelet activation, the importance of many others remains unclear. Furthermore, since ion channel proteins need only be present at low density to exert a significant influence on cell function, it is likely that additional important members of this protein superfamily are expressed in the platelet and to date remain undetected. For example, each platelet has been estimated to express only 150–300 functional P2X1 receptors (3), compared to approximately 50,000–80,000 copies of the integrin α_IIb_β_3_ (GPIIbIIIa) (4), yet this cation channel is a key contributor to thrombosis in small arteries and arterioles (5).

Proteomics is an important tool that has recently been used to identify ~4000 proteins in resting platelets from healthy donors (6, 7). Despite the large number of proteins reported, relatively few are ion channels or channel regulatory proteins (21 and 27 in these two studies). This result may reflect the difficulty of visualising proteins with multiple transmembrane domains, such as ion channels, using standard proteomics methods (8). Although anucleate, platelets contain mRNA, therefore an alternative approach to identify platelet proteins is via screening of the transcriptome. Over the last decade, gene expression microarray studies have reported an expanding list of transcripts within the platelet (9, 10). More recently, next-generation sequencing techniques have identified in excess of 9000 genes expressed in human platelets (11, 12). However, even when detection thresholds and correction for false discovery rates have been rigorously applied, qPCR is still required for validation of gene expression and estimation of transcript abundance from RNA-seq reads. Recent qPCR screening of platelet samples for all known voltage-gated K^+^ channel alpha subunits was a key step in the identification of Kv1.3 (gene symbol: *KCNA3*) as the pore-forming subunit of the voltage-dependent K^+^ conductance in platelets and megakaryocytes (13). In the present study, we have extended this approach to include 402 genes encoding ion channels and ion-channel-related proteins, with the aim of defining the platelet ion channelome. qPCR was also used to define the channelome of two human megakaryoblastic cell-lines (MEG-01 and CHRF-288–11), and a human erythroleukaemic/ megakaryocytic cell-line (HEL) (14) which are often used as surrogates for electrophysiological studies of the small, fragile platelet. This is the first study to focus specifically on determining the platelet ion channelome. The data highlight that platelets express mRNA transcripts encoding multiple ion channels and channel regulatory proteins with no known function in this cell type. The study also provides a reference atlas for future studies of this large family of proteins in megakaryocytic cell lines.

## Materials and methods

### Blood collection and platelet purification

Blood was collected with informed consent from healthy adult donors, as approved by the University of Leicester Human Biology Ethics committee (non-NHS). For each sample, 10 ml of whole blood was drawn into acid-citrate dextrose anticoagulant (ACD; 85 mM trisodium citrate, 78 mM citric acid and 111 mM glucose) and 1.5 ml of a platelet inhibitor cocktail was added immediately to prevent platelet activation (2 mM EDTA, 0.1 µM PGE_1_ and 0.3 mM acetylsalicylic acid). Platelet-rich plasma (PRP) was prepared by centrifugation for 20 minutes at room temperature at 150 × *g*, and the upper 80% was transferred to a clean tube to avoid transfer of leukocytes from the buffy coat. Contamination of PRP by leukocytes or platelet-leukocyte aggregates was further reduced by immunomagentic depletion of CD45+ve cells (Dynabeads^®^, Invitrogen, Paisley, UK). Platelets were then positively selected with Pan mouse IgG Dynabeads^®^ (Invitrogen) coated with mouse anti-human CD42b (Becton Dickinson, Oxford, UK). Following removal of the plasma supernatant, the bead-bound platelets were washed in sterile phosphate-buffered saline (PBS), and lysed in Lysis/Binding buffer (100 mM TrisHCl (pH 7.5); 500 mM LiCl; 10 mM EDTA; 1% LiDS; 5 mM DTT).

### Cell culture

MEG-01, CHRF-288–11, and HEL cell lines were obtained from the European Collection of Cell Cultures (ECACC, Salisbury, UK) and were cultured in RPMI-1640 medium containing 10% fetal calf serum (Invitrogen), supplemented with 100 µg/ml penicillin and 100 µg/ml streptomycin at 37oC in a humidified incubator with 5% CO_2_. A total of 4 × 10^6^ cells from each cell line were pelleted by centrifugation, washed in sterile PBS, and lysed in Lysis/Binding buffer.

### Quantitative real-time PCR

mRNA was directly isolated from all cells using Oligo-dT_25_ Dynabeads^®^ (Invitrogen). The mRNA-bound beads were washed in Tris buffer solution with or without Lithium Dodecyl-Sulfate (LiDS)(Buffer A– 10 mM TrisHCl (pH 7.5); 0.15 M LiCl; 1 mM EDTA; 0.1% LiDS; Buffer B– 10 mM TrisHCl (pH 7.5); 0.15 M LiCl; 1 mM EDTA), and resuspended in RNase-free water (Sigma, Dorset, UK). mRNA was reverse-transcribed using cloned AMV Reverse Transcriptase (Invitrogen). cDNA was stored at –80oC until required.

Quantitative-PCR analysis was carried out using QuantiFast SYBR green PCR (Qiagen, Manchester, UK) and QuantiTect primer assays (Qiagen) on a 7900HT sequence detection system analyser (ABI Prism, Invitrogen, UK). The genes included in the human ion channelome were identified through manual searches including PubMed (http://www.ncbi.nlm.nih.gov/pubmed); International Union of Basic and Clinical Pharmacology database (IUP HAR;http://www.iuphar-db.org/), Ingenuity Pathways Analysis (http://www.qiagen.com). All primer assays used in the study are listed in Suppl. Table 1 (available online at www.thrombosis-online.com). Each qPCR included amplification of the housekeeping gene *GAPDH* and cell-specific genetic markers CD45 (*PTPRC*)(leukocytes), *GYPA* (erythrocytes) and *GPIBB* (platelets), and cycling profiles were applied as per the manufacturer’s instructions. Melt curve analysis was performed to confirm specificity of all primers, and specificity was further validated by agarose gel electrophoresis as described elsewhere (15). Only qPCR amplification products matching the theoretical amplification size supplied by Qiagen were assigned as positive. The △Ct method was used to calculate ion channel gene expression levels, which were then normalised relative to expression of *GAPDH.* Results are shown as the mean relative gene expression ± SEM. The platelet channelome results are shown as the mean of data from four separate donors. Channelome results for cell-lines are based on three separate experiments per cell type.

## Results

### Optimisation of platelet purification

**Figure 1:**
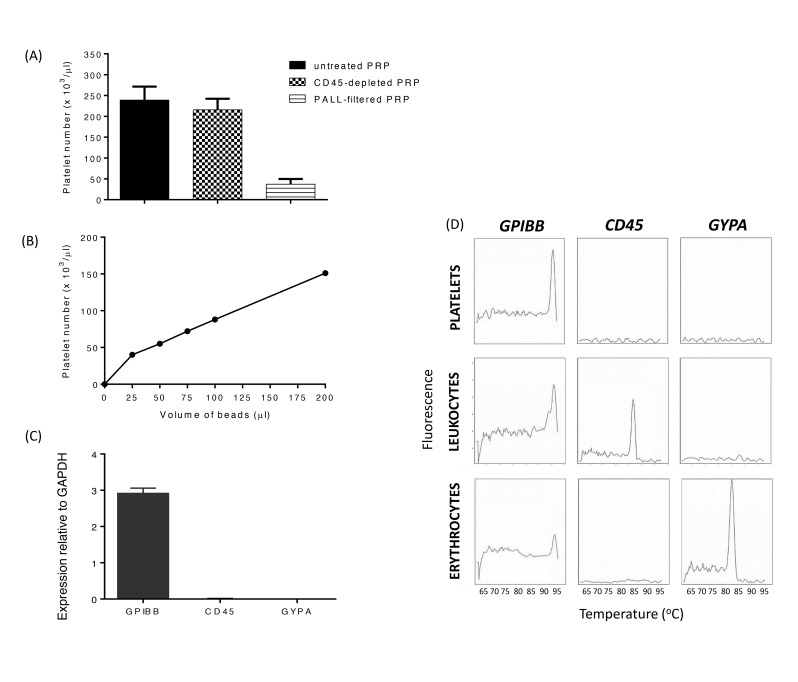
**Isolation of ultrapure platelet mRNA.** A) Leukocyte depletion using CD45-coated magnetic beads resulted in only minimal loss of platelets from PRP in comparison to leukocyte depletion using PALL filtration. B) Increasing volumes of CD42b-coated magnetic beads enabled positive selection of ~75% of platelets from 1.5 ml of PRP. C) To test for purity of platelet sample preparations, platelet mRNA was reverse transcribed and amplified for detection of cell-specific markers, *GPIBB* (platelets), *CD45* (*PTPRC*) (leukocytes) and *GYPA* (erythrocytes), and expressed relative to the endogenous reference gene *GAPDH*. D) Melt curve analysis for platelet (*GPIBB*), leukocyte (*CD45*) and erythrocyte (*GYPA*) in platelet sample preparations (top row) used for channelome screening, and in leukocyte (middle row) and erythrocyte (bottom row) sample preparations for comparison.

Whilst qPCR is recognised as one of the most sensitive methods for detection of expressed transcripts, previous studies have highlighted the low mRNA content of platelets and thus the possibility of compromised data due to contamination of mRNAs from other blood cells. Furthermore, mRNA can be rapidly degraded, which represents a problem for detection of genes expressed at low levels. Therefore, our initial aim was to improve upon previously reported approaches for platelet purification and cDNA library generation. A combination of inhibitors (see Methods) reduced the possibility of platelet activation leading to leukocyte-platelet aggregates. Although Pall leukocyte depletion filters represent a useful approach to remove leukocytes and possible leukocyte-platelet aggregates (15), we found that they substantially reduced the platelet yield (▶ Figure 1A). In contrast, leukocyte depletion using CD45-coated magnetic beads did not significantly affect platelet number (▶ Figure 1A). Following leukocyte depletion, the proportion of platelets positively recovered using anti-CD42b-coated magnetic beads increased with bead density (▶ Figure 1B), reaching ≈75% recovery at the level (200 µl of beads per 1.5 ml of PRP) used subsequently in this study. Following wash steps to remove plasma components, mRNA was directly extracted using OligodT_25_ beads and immediately reverse transcribed, thereby limiting the time available for mRNA degradation. qPCR of transcripts encoding cell-specific markers for platelets (*GPIBB*), leukocytes (*CD45*), and erythrocytes (*GYPA*) demonstrated the high purity of platelets in our preparations (▶ Figure 1C). Leukocyte and erythrocyte samples, obtained from the buffy coat and red cell pellets respectively, provided positive controls for the *CD45* and *GYPA* assays (▶ Figure 1D).

### Quantification of expression of the platelet channelome

qPCR screening of our human platelet cDNA library to identify the ‘channelome’ resulted in the detection of 84 ion channel or ion channel-related transcripts from a total of 402 genes. Of these, it was possible to provide a rank order of mean expression of 34 genes (▶ Table 1), with 31 of these genes being expressed in at least three of the four donors, and three (*CLCN4, FXYD1*, and *ANO10*) being quantifiable in two of the four donors. The remaining 50 ion channel transcripts were detected at lower levels, outside of the range of accurate, linear amplification for the qPCR primer assays, and were thus designated as being present only at trace levels (▶ Table 2). Throughout the results section, when referring to platelets, quantifiable genes are defined as GENE NAME (q) and those detected at trace levels as GENE NAME (t). Only 10 of the 84 transcripts detected (11.4%) have been functionally characterised in platelets or megakaryocytes [*ANO6* (alternatively named *TMEM16F*)(q), *P2XR1* (q), *KCNA3* (q), *CLIC1* (q), *ORAI1* (q), *PANX1* (q), *STIM1* (q), *TRPC6* (q), *ITPR1* (q) and *ITPR2* (q)], and have known agonist-evoked roles in haemostasis or thrombosis (2, 16). Seventy-five detected transcripts encoded pore-forming proteins and included 31 voltage-gated channels, 17 ligand-gated channels, and 23 other channel types, as classified by the IUPHAR database, whilst 13 transcripts encoded regulatory proteins or sub-units. These families are discussed further below.

### The channelomes of three leukaemic myeloid cell lines

**Figure 2:**
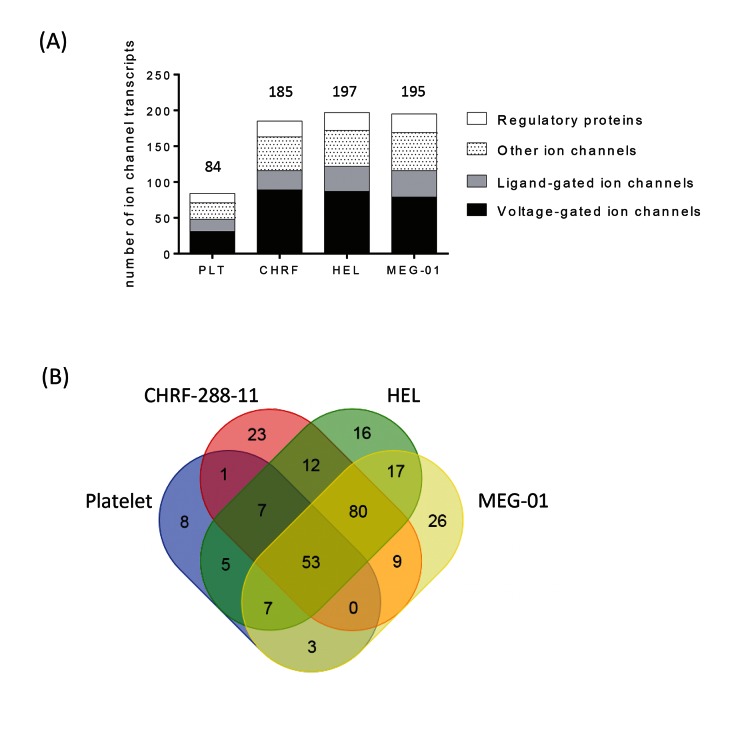
**The channelome of platelets and cell-lines.** A) Classification of ion channel-related transcripts in platelets and cell lines. The number of ion channel transcripts detected is shown for each cell type, and is further divided into channel type or regulatory protein according to the IUPHAR and UniProt classifications. B) Venn diagram depicting the distribution and overlap of ion channel-related transcripts between platelets, and the cell lines CHRF-288–11, HEL and MEG-01.

A greater number of ion channel transcripts were detected in all three cell lines compared to the platelet (185, 197 and 195 for CHRF-288–11, HEL and MEG-01 respectively, vs 84 for the platelet) (▶ Figure 2A). Differences in abundance of GAPDH transcripts make it difficult to compare the relative expression levels *per se* between the cell lines and the platelets. However, it is clear that the ion channel expression profiles in these cell lines are very different to that of the platelet, with only 53 transcripts being detected in all four of the cell types (▶ Figure 2B). ▶ Table 3 lists the top 20 most abundantly expressed transcripts detected in the cell lines, and a complete list of the relative expression for all channel transcripts included in the study is provided in the Suppl. Table 1 (available online at www.thrombosis-online.com).

### Chloride channels show prominent expression in the platelet

Nine of the quantifiable platelet transcripts, including the four most abundant, encode chloride-permeable ion channels. These include four members of the Anoctamin/TMEM16 family and one member of the Bestrophin family (*BEST3*)(q). A crucial role for *ANO6* (q), the second most abundantly expressed transcript in platelets, has been firmly established in calcium-dependent exposure of phosphatidylserine (PS) on the platelet surface and may contribute to regulation of platelet volume and generation of platelet microparticles in response to haemostatic agonists (16–19). The exact mechanisms whereby *ANO6* contributes to these processes remains to be elucidated. In Ano6-deficient mice, significant platelet scramblase activity remains (16), and the underlying proteins for this residual response are not known. Since chloride channel blockers have been reported to block PS exposure in platelets (20), the anion-permeable channels in the platelet channelome list provide a number of possible candidates; however, it is currently unclear whether the other platelet anoctamin channels detected, *ANO2*(q), *ANO8*(t) and *ANO10*(q), have scramblase activity (17). Interestingly, *ANO2*, *ANO6*, *ANO8* and *ANO10* were recently identified in murine platelets with the same order of expression as the present study (21). Our channelome analysis demonstrates that whereas detection of *ANO2* was platelet-specific, *ANO6, ANO8* and *ANO10* were also expressed in all three cell lines (Suppl. Table 1, available online at www.thrombosis-online.com).

We also detected platelet transcripts for the chloride intracellular channels *CLIC1*(q), *CLIC3*(t) and *CLIC4*(q). CLIC proteins can exist in both soluble and membrane-inserted forms and also exhibit enzymatic activity (22). While there have been no platelet studies identifying the role of CLIC3 or the most abundantly expressed transcript, CLIC4, absence of CLIC1 in mice has been associated with a prolonged bleeding time and a reduced platelet response to ADP (23). These channels may regulate platelet function by affecting ion flow through intracellular organellar membranes; however, they may also become inserted into the plasma membrane following platelet activation, as shown for CLIC1 in platelets (23). We additionally detected transcripts from the CLCN (CIC) family which have been associated with endosomal acidification and trafficking of vesicles (24), finding *CLCN3*(q), *CLCN4*(q), *CLCN6*(t) and *CLCN7*(t) in platelets and in all three cell lines. Voltage-dependent anion channels (VDACs) were also expressed at high levels in platelets and all cell lines. VDACs are located on the outer mitochondrial membrane, regulating the exchange of metabolites between the cytosol and the mitochondrial intermembrane space. Mitochondrial ion channels are key components of the intrinsic apoptosis pathway and contribute to platelet lifespan and cancer cell survival; however, the role of VDACs in platelet biology is unknown. We detected high expression of isoforms *VDAC2*(q) and *VDAC3*(q) in platelets, and all three isoforms were expressed in the cell lines. Several of the uncharacterised platelet chloride channels, such as *CLIC4*, *CLIC1*, *VDAC2* and *VDAC3*, were also amongst the most prevalent transcripts in the CHRF, HEL and MEG-01 cell lines, thus providing an alternative system for their study.

### Potassium channels

A total of 29 ion channel genes expressed in the platelet encode proteins involved in the transport of potassium ions. Nine of these are known to encode pore-forming domains and 19 to be ancillary subunits that modulate K^+^ channel function. The most abundantly expressed pore-forming potassium channel transcript was for the voltage-gated, shaker-related, pore-forming alpha subunit, Kv1.3 (*KCNA3*)(q), which we have previously reported to be responsible for the major potassium conductance of the platelet and maintenance of the resting membrane potential (13). High platelet mRNA levels were also detected for *KCNK6*(q), a two-pore potassium channel. This channel is widely expressed, and has been reported at the protein level in platelets (6, 7); however, it has not been observed in patch clamp studies, and its role in platelet function remains to be determined. KCa1.1 (*KCNMA1*)(t), a large conductance calcium-activated potassium channel not previously characterised in platelets, and three regulatory subunits of *KCNMA1* were also detected (*KCNMB1*(q), *KCNMB3*(t) and *KCNMB4*(q)). Amongst the other K^+^ channel regulatory subunit transcripts detected were the *KCNAB1*(t), *KCNAB2*(t) and *KCNAB3*(t) voltage-gated shaker-related subunits, also *KCNE2*(t) (Mirp1) and *KCNE3*(q) (Mirp2) from the Isk-related family, and potassium family regulatory protein (*KCNRG*)(t), which has been reported to suppress voltage-gated K^+^ channel currents in *Xenopus* oocytes (25). Additionally, we detected nine transcripts encoding potassium channel tetramerisation domain-containing proteins (KCTDs) (▶ Table 1 and ▶ Table 2). Very little is currently known about this large family of proteins, although some of these proteins have been reported to interact with GABAB GPCRs, resulting in modulation of receptor sensitisation (26). Further studies are required to determine the possible roles these regulatory proteins may play in platelet function.

### Calcium-permeable ion channels

Elevation of cytosolic calcium is essential for activation of most platelet functional responses. Amongst the known Ca^2+^-permeable channels that we detected in platelets were *P2RX1*(q), *ORAI1*(q), *TRPC6*(q), and the inositol 1,4,5-trisphosphate (IP_3_) receptor isoforms, *ITPR1*(q) and *ITPR2*(q). This provides further evidence that TRPC6 is the main member of the TRP family of non-selective cation channels expressed in platelets, as previously suggested (27). Despite several reports of TRPC3 function in platelets (28, 29), we did not detect transcripts for *TRPC3* mRNA, although it was detectable in all three cell-lines. Trace levels of *TRPP1*(t) (also known as *PKD1* and polycystin) channels were also detected in platelets. We have previously reported detection and function of TRPM7 in murine primary megakaryocytes (30), and in agreement with this we detected *TRPM7* in both megakaryocytic cell-lines; however, we did not detect *TRPM7* in platelets. A wider range of TRP channels were detected in the leukaemic lines, with *TRPV2* the most highly expressed TRP channel transcript in all three cell lines. This heat- and mechanically-activated channel was not observed in the platelet; however, consistent with this study, it has been implicated in haematologic malignancies (31).

It is well established that platelet store-operated calcium entry (SOCE) is mediated through activation of channels formed by Orai1 in response to STIM1-mediated detection of reduced calcium content of the dense tubular system (DTS) (32, 33). Abundant *ORAI1*(q) transcripts were detected in platelets and all cell lines, whereas only trace levels of *ORAI2*(t) mRNA were found in all cell types. *ORAI3* transcripts were quantifiable in all three cell lines but were not detected in the platelet. Transcripts for the calcium sensors *STIM1*(q) and *STIM2*(t) were present in platelets and all cell lines. Studies in *Stim2*-KO mice suggest there is normal platelet function and calcium homeostasis in response to GPVI- and thrombin-dependent activation (34). Interestingly, STIM2 has been shown to act as a regulator of basal calcium concentrations in the endoplasmic reticulum (ER) of HeLa cells (35). We also detected *TMEM109*(q), an organellar membrane protein which may serve as a counterion transport mechanism during Ca^2+^ store release and has been associated with cell death in murine thymocytes (36).

Agonist binding to platelet surface GPCRs and tyrosine kinasecoupled receptors results in generation of IP_3_ and thus activation of calcium-permeable IP_3_ receptors (IP_3_Rs); however, to date the relative expression of the three IP_3_R isoforms in the platelet remains unclear. We detected type 1 (*ITPR1*)(q) and type 2 (*ITPR2*)(q) IP_3_R mRNAs in platelets, although surprisingly a greater number of transcripts of type 2 IP_3_Rs were detected. An alternative pathway of calcium mobilisation from intracellular stores has been suggested to occur via two-pore channels, TPCN1 and TPCN2. Whereas both isoforms were detected in the three cell lines, only *TPCN1*(t) was detected in platelets. These calcium-permeable ion channels are sensitive to pyridine nucleotides, NAADP, and insert into lysosomal organelles, releasing calcium into the cytoplasm from the acidic stores (37); however, further study is needed to demonstrate that this calcium-activated calcium release channel is functional in the platelet. mRNAs encoding P2X1 receptors (*P2RX1*)(q) were the fifth most abundantly expressed platelet ion channel transcript. This ATP-gated non-selective cation channel has been well characterised in platelets and shown to contribute to arterial thrombosis (2, 5). We found only trace detection of *P2RX4*(t) and *P2RX6*(t) transcripts, in agreement with previous work showing that P2X1 is the dominant ATP-gated P2X receptor of the platelet. *P2RX1* was also expressed in the HEL and MEG-01 cell lines as found previously (38, 39), and the cell lines also expressed *P2RX4*, *P2RX5* and *P2RX7*.

In addition to ATP, platelets also secrete the neurotransmitters glutamate, serotonin and acetylcholine. Functional NMDA, AMPA and kainite ionotropic glutamate receptors have been reported in the platelet and megakaryocyte (2, 40, 41). We detected components of kainate (*GRIK4*(t), *GRIK5*(t)), NMDA (*GRIN2D*)(t) receptors, and *GRINA* (protein lifeguard 1/NMDA receptor associated protein 1)(t), but not AMPA receptor transcripts in platelets. Whereas it has been established that activation of the platelet GPCR serotonin receptor 5HTR2A results in calcium mobilisation (42), the presence and function of 5HTR3 ion channel family members remains less certain. Our qPCR screen included the 5HTR3 ion channel family of genes, i.e. all known serotonin-gated ion channels, finding no detectable transcripts in human platelets, but quantifiable levels of HTR3D in CHRF, and trace detection of the same gene in HEL and MEG-01 cells. It has been suggested that acetylcholine potentiates the platelet response to ADP and thromboxane A2, influencing early aggregation (2). In the present study we detected mRNA encoding the calcium-permeable α2 nicotinic cholinergic receptor (*CHRNA2*)(q) only in platelets, whereas the α5 transcript (*CHRNA5*) was detected only in the cell lines. Other ligand-gated channels detected included GABAA-receptor subunits (*GABRD*(t) and *GABRR2*(t)) and glycine receptors (α-subunits *GLRA2*(t) and *GLRA4*(4), and β-sub-unit *GLRB*(t)). Ligand-binding of glycine may result in the influx of chloride ions, thereby regulating the platelet membrane potential (43).

### Possible roles of other detected ion channels in the platelet

Gap junction-mediated intercellular communication allows the brief transfer of small molecules (up to ≈1 kDa). Twenty members of the connexin family have been reported in mammals. We detected Cxn 37 (*GJA4*)(t), which is known to contribute to platelet function (44, 45), and also mRNAs for Cxn 30.3 (*GJB4*)(t) and Cxn 31.1 (*GJB5*)(t), not previously reported in platelets. A wider range of connexin transcripts were detected in the cell-lines but only at trace levels. Although similar in structure to connexins, pannexins do not form gap junctions (46); however, the channels (sometimes referred to as hemi-channels) allow rapid permeation of molecules <1kDa in size. Three pannexin (PANX) hemi-channel subtypes exist in mammals, but platelets and MEG-01 cells only expressed *PANX1*(q). Studies in our laboratory have recently demonstrated functional surface pannexin-1 in platelets, demonstrating a mechanism for non-vesicular release of ATP (47). CHRF expressed *PANX2*, and HEL expressed all three PANX transcripts. Interestingly, *LRRC8B*(q) transcripts were also detected in platelets. The LRRC8 family of proteins (Leucine-rich repeat containing protein 8), share sequence and structural homology with the pannexin family (48), and have recently been proposed to be components of a volume-regulated ion channel (VRAC) in human embryonic kidney (HEK) cells (49).

Regulation of cell volume is crucial to maintain the integrity of the cell membrane. One ion channel family, as yet uncharacterised in platelets or megakaryocytes, is the aquaporins. We detected *AQP10*(q), encoding an aqua-glyceroporin, and low levels of *AQP1*(t) in platelets. These transcripts were also detected in the cell lines, which additionally expressed *AQP3* and *AQP11*. An increase in cell volume and size results in stretching of the plasma membrane, and this change in membrane tension is detected by mechanosensitive proteins. To date there have been no mechanosensitive ion channels identified in platelets. This study found low levels of transcripts encoding *PIEZO1* (*FAM38A*)(t), reported to be a calcium-permeable mechanically activated cation channel involved in homeostasis of erythrocyte cell volume (50).

In addition to their haemostatic function, platelets also contribute to innate immunity; however, the identities and importance of specific ion channels that contribute to this response remains to be determined. The voltage-gated proton channel, *HVCN1*(t), detected in platelets and all cell lines, has an established role in supporting superoxide generation via NADPH oxidase, which is known to take place in platelets. Additional contributions to cellular responses have emerged for HVCN1 in recent years, including modulation of immune receptor signalling in B lymphocytes (51). Detection of other channel components in platelets included trace levels of the ion conducting pore-forming alpha-subunit *SCN5A* (Nav1.5)(t), and two beta subunits, *SCN1B* (beta-1)(t) and *SCNB3* (beta-3)(t). Generally associated with expression in cardiac muscle, this sodium channel has also been reported in human monocyte-derived macrophages, regulating endosomal pH and phagocytosis through attenuation of localised calcium oscillations during exposure to bacterial lipopolysaccharides (52).

## Discussion

Although platelets are known to express a number of ion channels with important functional roles (5, 32, 33, 38, 53, 54), we hypothesised that additional members of this superfamily of membrane proteins are expressed and may not have been detected in previous studies of the whole platelet proteome or transcriptome. This proposal was based on two main facts: firstly, ion channels need only be expressed at low copy number to influence cell function, and secondly, platelets contain low levels of RNA. Therefore, detection of ion channels amongst more abundantly expressed proteins or transcripts may be problematic. Proteomic and transcriptomic approaches have their advantages and disadvantages, and in addition, there is disagreement as to the degree of correlation between the transcriptome and proteome (7, 9, 10). Comparison of one RNA-seq study (55), with the findings of the proteomic report from Burkhart et al. (7) suggests that whereas the platelet transcriptome has a high inter-individual correlation, a significant number of transcripts are not translated (55). In contrast, Rowley et al. (56) suggest a much higher correlation between proteome and transcriptome. In support of the latter, a frequency density distribution analysis of platelet transcriptomic and protemic data reported that they are almost identical (57). The on-going debate highlights that there are many factors to be borne in mind when comparing proteomic and transcriptomic datasets, including donor variation, ID mapping, methodological and statistical handling differences.

Whilst recent proteomic studies have made a significant contribution to the characterisation of the platelet proteome (6, 7), methods used within the proteomic process may result in the under-representation of transmembrane proteins such as ion channels. One example of this is a recent study of the human proteome which characterised the proteomic signature of several tissues, that reported only five ion channels in the platelet (58). In addition, a common limitation of both proteomic and transcriptomic array studies is the difficultyof directly quantifying low abundance proteins or mRNA transcripts, and also the masking of weak signals by the products of highly expressed genes. Therefore, in this study we have used qPCR and focused only on ion channel-related targets, an approach that we have used previously to successfully identify *KCNA3* (Kv1.3) (13) and *PANX1* (Pannexin-1) (47) ion channel transcripts in human platelets. These early studies have demonstrated an important role for Kv1.3 in maintenance of the platelet resting membrane potential, and for Pannexin-1 in the release of non-vesicular ATP leading to amplification of calcium influx. The results of our ‘channelome’ study of ion channel-related transcripts compare favourably with a RNA-seq study of the whole human platelet transcriptome (11), reporting an overlap in detection of 61 ion channel–related mRNA transcripts from our ion channel database; with *ANO6* (*TMEM16F*) being significantly expressed in both studies. The most abundantly expressed transcripts in our study included nine channels previously reported and at least partly characterised in platelets, and the order of their expression correlates closely both with estimations of relative channel density reported in patch clamp recordings, and correlates at least partly with the estimated copy number determined by proteomic quantification (7) (▶ Table 4).

One of the major sources of platelet mRNA is its precursor cell, the megakaryocyte (MK). During MK maturation, mRNA is packaged into granules and transported into the proplatelet tips and therefore is present in the newly formed platelet. A second possible source of platelet mRNA is that which has been taken up by the platelet, for example from microvesicles that are elevated in the circulation in a range of disease states. The platelets used in this study were collected from healthy donors with no known disease, and therefore the mRNA analysed is likely to reflect a transcriptome generally found in platelets within the circulation of healthy individuals, the source of which is predominantly from the megakaryocyte. Therefore, decay of mRNA, together with the relatively low abundance of mRNA in comparison to other peripheral blood cells must be considered as a possible limitation to transcriptomic study results. Indeed, our previous whole cell patch clamp recordings show that each human platelet possesses only 5–7 functional medium conductance Ca^2+-^activated K^+^ channels (KCa3.1, the Gardos channel, gene name *KCNN4*) (59), but we were unable to detect this extremely low abundance transcript in platelets. In contrast, it was observed in all three leukaemic cell lines (see below), including HEL cells, where robust currents through this channel have been reported in patch clamp studies (60). Interestingly there were 53 ion channel-related transcripts that were only expressed in the three cell lines and not in the platelet, these included *TRPC1*, *TRPC3*, and *TRPM7*, therefore it is possible that some of these channels are also so few in number that they are below the level of detection at the transcriptomic level in this study. Conversely, there were six transcripts detected in platelets that were not present in any of the cell-lines. Of this group, the most abundantly expressed transcript was *ANO2* (alternatively named *TMEM16B*), encoding the second member of the Calcium-activated chloride channels, Anoctamin-2. There are several possible explanations as to why these transcripts were only detected in the platelets and not in the cell lines; including deletion or insertion of additional transcripts during continual culture of cell lines, or reflection of a genotype/phenotype from their cancer cell origin. Of note, Kv1.3 (*KCNA3*) was absent in CHRF and HEL cell lines and detected only at trace levels in MEG-01. This agrees with a previous report that voltage-dependent K^+^ currents are absent or greatly reduced in megakaryocytic cell lines and in megakaryocytes from patients undergoing treatment for myelogenous leukaemia (61). These findings suggest that although key cell lines provide a useful alternative tool for functional studies of the platelet, the surrogate cell must be selected with care and results must be interpreted cautiously. Therefore, our database of ion channel transcripts that are present in megakaryocytic and erythroleukaemic cell-lines provides a useful resource for selection of a suitable surrogate cell for studies of megakaryocyte ion channel function and for the study of ion channels in cancer.

**What is known about this topic?**

- Ion channels have crucial roles in all cell types and represent important therapeutic targets.
- Approximately 20 ion channel subtypes have been reported in human platelets, and it is likely that platelets express additional members of this large family of membrane proteins.
- Megakaryocytic cell lines are frequently used as a tool to study the anuclear platelet; however, there has been no comprehensive study to compare their ion channel profile.

**What does this paper add?**

- This is the first study to define the human platelet ion channelome. It highlights the prominent expression of a number of ion channels that have not previously been identified in platelets and that have as yet unknown functional roles in platelet physiology and pathophysiology.
- The data provides an atlas of ion channel expression in cell-lines of megakaryoblastic/erythroleukaemic origin which may aid the selection of an appropriate surrogate cell for electrophysiological study of platelet ion channels.
- The cell-line channelome data also provides a resource that may facilitate future studies of ion channel expression in myeloid leukaemia and the identification of new therapeutic targets.

**Figure 3:**
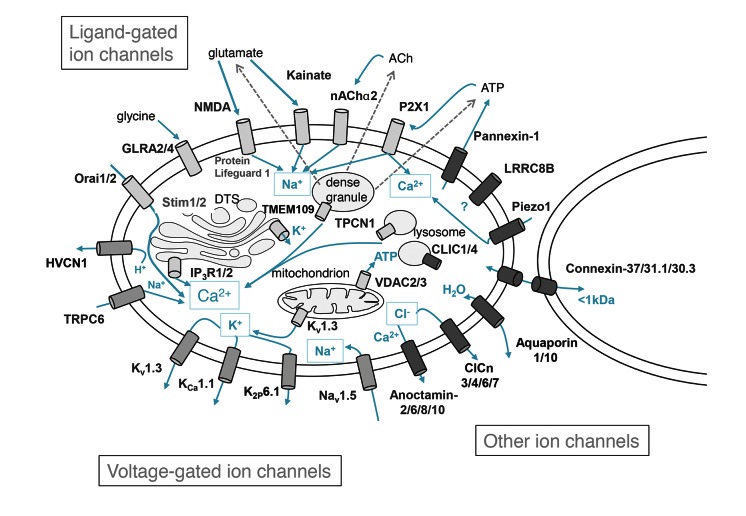
The human platelet channelome. A schematic summary of ion channels detected by quantitative-PCR analysis of ultrapure platelet sample preparations, classified into ligand-gated ion channels (light grey), voltage-gated ion channels (medium grey), and other ion channels (dark grey).

In conclusion, the current study was designed to characterise the gene expression profile of the human platelet channelome. The results show that platelets express a range of different ion channels (▶ Figure 3), many of which have undetermined roles in this cell type. Future studies of such proteins may prove beneficial given the widespread use of ion channels as therapeutic targets and the key roles of platelets in a range of physiological and pathophysiologic processes. Although megakaryocytic cell lines have proven a useful tool for study of certain platelet proteins, the channelome datasets also highlight that such cells must be selected carefully in surrogate studies of platelet ion channels. Future investigations of the differences in ion channel expression between platelets and related leukaemic cell lines may also improve our understanding of cellular events that contribute to myeloid leukaemia.

## Supplementary Material

Supplementary Data Table 1: Quantitative PCR measurements of all ion channel-related transcripts in human platelets and three megakaryocytic cell lines

## Figures and Tables

**Table 1:** **Quantified ion channel related transcripts in human platelets.** Data represent expression of platelet mRNA transcripts relative to endogenous control. Results are expressed as mean values ± SEM. Reported platelet function detected in: hP, human platelets; hD, human disease; mKO, murine knock-out; mTG, murine transgenic. MP, microparticle.

Gene symbol	Full name	Relative expression (±SEM)	Reported platelet function?
CLIC4	Chloride intracellular channel 4	2.759 ± 0.555	No
ANO6	Anoctamin 6 (TMEM16F)	2.020 ± 0.632	Scramblase activity hP, MP production hP mKO, Scott syndrome hD, increased bleeding time mKO (16–18, 21, 62)
VDAC3	Voltage-dependent anion channel 3	0.645 ± 0.235	No
CLCN3	Voltage-sensitive chloride channel 3	0.581 ± 0.161	No
P2RX1	Purinergic receptor P2X, ligand-gated ion channel, 1	0.397 ± 0.202	ATP-gated Ca^2+^ influx hP, arterial thrombosis hP mTG (3, 5, 38, 63)
FXYD5	FXYD domain-containing ion transport regulator 5, dysadherin	0.345 ± 0.107	No
KCNA3	Voltage-gated potassium channel alpha subunit, Kv1.3	0.249 ± 0.062	Membrane potential hP, increased platelet count mKO (13)
CLIC1	Chloride intracellular channel 1	0.241 ± 0.041	Role in ADP-evoked signalling mKO (23)
LRRC8B	Leucine-rich repeat-containing protein 8B	0.202 ± 0.088	No
ORAI1	ORAI calcium release-activated calcium modulator 1	0.124 ± 0.028	Store Operated Ca^2+^ Entry (SOCE) hP mKO, impaired thrombus formation mKO (32, 33)
PANX1	Pannexin 1	0.113 ± 0.039	ATP release hP mKO (47, 64)
KCNE3	Potassium voltage-gated channel, Isk-related family member 3, MiRP2	0.092 ± 0.019	No
ITPR2	Inositol 1,4,5-triphosphate receptor, type 2	0.088 ± 0.040	Agonist-evoked Ca^2+^ mobilisation hP (2, 65)
STIM1	Stromal interaction molecule 1	0.086 ± 0.023	Ca^2+^ sensor for SOCE hP mKO, impaired thrombus formation mKO (2, 32, 66, 67)
VDAC2	Voltage-dependent anion channel 2	0.053 ± 0.016	No
KCTD20	Potassium channel tetramerisation domain-containing protein 21	0.051 ± 0.005	No
TRPC6	Transient receptor potential cation channel, subfamily C, member 6	0.050 ± 0.030	Ca^2+^ entry, haemostasis and thrombogenesis hP mKO (2, 27, 28, 68, 69)
ANO2	Anoctamin 2 (TMEM16B)	0.037 ± 0.007	No
KCTD10	Potassium channel tetramerisation domain-containing protein 10	0.036 ± 0.011	No
KCNK6	Potassium channel, subfamily K, member 6; K2p6.1	0.035 ± 0.010	No
AQP10	Aquaporin 10	0.034 ± 0.014	No
CLCN4	Voltage-sensitive chloride channel 4	0.033 ± 0.004	No
FXYD1	FXYD domain-containing ion transport regulator 1, phospholemman	0.025 ± 0.012	No
KCNMB1	Calcium-activated potassium channel subfamily M, K(VCA)beta-1	0.025 ± 0.011	No
BEST3	Bestrophin-3, Vitelliform macular dystrophy 2-like protein-3	0.025 ± 0.002	No
CHRNA2	Cholinergic receptor, nicotinic, alpha 2	0.023 ± 0.007	No
KCTD18	Potassium channel tetramerisation domain-containing protein 18	0.022 ± 0.003	No
KCTD2	Potassium channel tetramerisation domain-containing protein 2	0.016 ± 0.003	No
KCTD13	Potassium channel tetramerisation domain-containing protein 13	0.016 ± 0.005	No
ITPR1	Inositol 1,4,5-triphosphate receptor, type 1	0.015 ± 0.002	Agonist-evoked Ca^2+^ mobilisation hP (2, 65, 70)
ANO10	Anoctamin 10	0.011 ± 0.002	No
KCNMB4	Calcium-activated potassium channel subunit beta-4	0.009 ± 0.003	No
TMEM109	Transmembrane protein 109; Mitsugumin-23.	0.007 ± 0.001	No
KCTD9	Potassium channel tetramerisation domain-containing protein 9	0.004 ± 0.001	No

**Table 2:** Ion channel-related transcripts detected at trace levels in human platelets.

Gene symbol	Protein name	Gene symbol	Protein name
ANO8	Anoctamin 8	KCNE2	Mirp1
AQP1	Aquaporin 1 (Colton blood group)	KCNG4	Modifier/silencer; Kv6.4
CACNB1	Calcium channel, voltage-dependent, beta 1 subunit	KCNK1	Potassium channel, subfamily K, member 1; K2p1.1, TWIK-1
CATSPER1	CatSper1; cation channel sperm-associated protein-1	KCNK17	Potassium channel, subfamily K, member 17; K2p17.1, TASK4
CLCN6	Chloride channel 6	KCNMA1	Potassium large conductance calcium-activated channel, subfamily M, alpha member 1
CLCN7	Chloride channel 7	KCNMB3	Calcium-activated potassium channel subunit beta-3
CLIC3	Chloride intracellular channel 3	KCNN3	Potassium intermediate/small conductance calcium-activated channel, subfamily N, member 3; KCa2.3
FXYD7	FXYD domain-containing ion transport regulator 7	KCNQ4	Non-inactivating voltage-gated; Kv7.4
GABRD	Gamma-aminobutyric acid (GABA) A receptor, delta	KCNRG	Potassium channel regulatory protein
GABRR2	Gamma-aminobutyric acid (GABA) A receptor, beta 2	KCNT2	Potassium channel, subfamily T, member 2
GJA4	Gap junction protein, alpha 4, 37kDa	KCTD11	Potassium channel tetramerization domain-containing protein 11
GJB4	Gap junction protein, beta 4, 30.3kDa	KCTD5	Potassium channel tetramerization domain-containing protein 5
GJB5	Gap junction protein, beta 5, 31.1kDa	KCTD7	Potassium channel tetramerization domain-containing protein 7
GLRA2	Glycine receptor, alpha 2	ORAI2	ORAI calcium release-activated calcium modulator 2
GLRA4	Glycine receptor, alpha 4	P2RX4	Purinergic receptor P2X, ligand-gated ion channel, 4
GLRB	Glycine receptor, beta	P2RX6	Purinergic receptor P2X, ligand-gated ion channel, 6
GRIK4	Glutamate receptor, ionotropic, kainate 4	PIEZO1	Fam38A; Piezo-type mechanosensitive ion channel component
GRIK5	Glutamate receptor, ionotropic, kainate 5	PKD1	Polycystin-1; TRPP1
GRIN2D	Glutamate receptor, ionotropic, N-methyl D-aspartate	SCN1B	Sodium channel, voltage-gated, type I, beta
GRINA	Protein lifeguard 1; Glutamate [NMDA] receptor-associated protein 1	SCN3B	Sodium channel, voltage-gated, type III, beta subunit
HVCN1	Hydrogen voltage-gated channel 1	SCN5A	Sodium channel, voltage-gated, type V, alpha subunit; Nav1.5
KCNA2	Shaker-related non-inactivating delayed rectifier alpha, Kv1.2	SCNN1A	Sodium channel, nonvoltage-gated 1 alpha
KCNAB1	Beta subunit shaker-related, Kvbeta1	STIM2	Stromal interaction molecule 2
KCNAB2	Beta subunit shaker-related, Kvbeta2	STX1B	Syntaxin-1B
KCNAB3	Beta subunit shaker-related, Kvbeta3	TPCN1	Two pore segment channel 1

**Table 3:** **Most abundant ion channel-related transcripts in megakaryoblastic and megakaryocytic cell lines.** Data represent expression of mRNA transcripts relative to endogenous control. Results are expressed as mean values (see Suppl. Table 1 for SEM, available online at www.thrombosis-online.com). nd, not detected; td, trace detection.

Gene symbol	Full name	Relative expression		
		CHRF	HEL	MEG-01
CLIC1	Chloride intracellular channel 1	0.2338	0.0662	0.0827
CLNS1A	Chloride conductance, nucleotide sensitive protein 1A	0.1066	0.0683	0.0750
CLIC4	Chloride intracellular channel 4	0.0606	0.0962	0.0503
VDAC2	Voltage-dependent anion channel 2	0.0944	0.0611	0.0315
VDAC3	Voltage-dependent anion channel 3	0.0465	0.0412	0.0859
FXYD5	FXYD domain-containing ion transport regulator 5; dysadherin	0.0425	0.0675	0.0294
TOMM40	Mitochondrial import receptor subunit homolog; Protein haymaker, p38.5	0.0305	0.0300	0.0568
CLIC2	Chloride intracellular channel 2	0.0005	0.0155	0.0499
CLCA1	CLCA family member 1, chloride channel regulator	nd	nd	0.0449
GRINA	Protein lifeguard 1; Glutamate [NMDA] receptor-associated protein 1	0.0078	0.0276	0.0254
PKD1	Polycystin-1; TRPP1	0.0262	0.0067	0.0073
VDAC1	Voltage-dependent anion channel 1; porin	0.0021	0.0022	0.0245
KCTD15	Potassium channel tetramerization domain-containing protein 15	0.0007	0.0210	0.0116
KCNK17	Potassium channel, subfamily K, member 17; K2p17.1	0.0181	0.0041	td
GJA4	Gap junction protein, alpha 4, 37kDa	0.0123	0.0013	td
GJA5	Gap junction protein, alpha 5, 40kDa	0.0004	0.0015	td
TMEM38B	TRIC type sub-channel B	0.0112	0.0087	0.0118
PIEZO1	Piezo-type mechanosensitive ion channel component; Fam38A	0.0051	0.0110	0.0088
KCNE3	Potassium voltage-gated channel, Isk-related family, member 3; MiRP2	0.0057	0.0105	0.0008
KCNN4	Potassium intermediate/small conductance calcium-activated channel, subfamily N, member 4; KCa3.1	0.0011	0.0047	0.0104

**Table 4:** **Estimated channel densities per platelet: electrophysiological and proteomic quantification.** MK, megakaryocyte; Plt, platelet; Cm, circumference; pS, picoSiemens; pA, picoamperes; mV, millivolts.

Channel gene name	Channel protein name	Patch clamp configuration	Maximum current or conductance	Single channel current or conductance	Estimated channels per platelet (electrophysiological quantification)	Reference	Estimated channels per platelet (Proteomic quantification (7))
*ANO6*	Anoctamin-6 TMEM16F	MK inside-out	100pS (+80mV)	0.45pS (+80mV)	≈1100	16	4000
*KCNA3*	Purinergic receptor P2X, ligand-gated ion channel 1	Plt whole-cell	106pA (-70mV)	0.71pA (-70mV)	≈150 functional; ≈750 total	2	1400
*KCNA3*	Voltage-gated potassium channel alpha subunit, Kv1.3	Plt whole cell	~100pA (+40mV)	0.35pA (-40mV)	≈285	71	<500
*ORAI1*	ORAI calcium release-activated calcium modulator 1	MK whole-cell (MK Cm is 1850 times Plt Cm)	100pA (-80mV)	1.4 fA (-80mV)	≈40	32	1700
*TRPC6*	Transient Receptor Potential cation channel, subfamily C, member 6	MK whole cell (predicted to be TRPC6)	≈50 pA (-60mV)	1.7 pA (-60mV)	1	30	1100
*CLIC1,* *CLIC4* *ITPR1* *ITPR2*	Chloride intracellular channel 1 Chloride intracellular channel 4 Inositol 1,4,5-triphosphate receptor, type 1 Inositol 1,4,5-triphosphate receptor, type 2	Organellar channels	Data not available				44000 16500 2400 1700
