# Supplementary material for: Transcriptomic Analysis of the Ion Channelome of Human Platelets and Megakaryocytic Cell Lines
Source: Thromb Haemost. 2016 Jun 9;116(2):272–84. doi: 10.1160/TH15-11-0891 (PMC5080539; doi:10.1160/TH15-11-0891)
Supplement: Supplementary Data Table 1: Quantitative PCR measurements of all ion channel-related transcripts in human platelets and three megakaryocytic cell lines [file TH-116-02-15110891-s001.pdf]

Supplementary Data Table 1

Quantitative PCR measurements of all ion channel-related transcripts in human platelets and three megakaryocytic cell lines

GENE NAME	PLT	SEM	CHRF	SEM	HEL	SEM	MEG-01	SEM	QuantiTect primer assay ID
ABCC8	n.d.		n.d.		n.d.		n.d.		QT00010605
ABCC9	n.d.		n.d.		n.d.		n.d.		QT00068607
ACCN1	n.d.		n.d.		n.d.		n.d.		QT00060830
ACCN2	n.d.		0.00012	4.93E-05	0.00200	9.99E-05	0.00046	4.48E-05	QT00042231
ACCN3	n.d.		0.00015	2.14E-05	0.00022	1.03E-05	trace		QT02310581
ACCN4	n.d.		0.00650	7.25E-04	0.00901	9.91E-04	0.00174	2.05E-05	QT00015134
ACCN5	n.d.		n.d.		n.d.		n.d.		QT00049294
ANO1	n.d.		n.d.		n.d.		n.d.		QT00076013
ANO10	0.01121	1.61E-03	0.00208	2.72E-04	0.00453	1.10E-04	0.00258	1.76E-04	QT01192835
ANO2	0.03692	6.03E-03	n.d.		n.d.		n.d.		QT00026656
ANO3	n.d.		n.d.		n.d.		n.d.		QT00015743
ANO4	n.d.		n.d.		n.d.		n.d.		QT00011214
ANO5	n.d.		n.d.		n.d.		trace		QT00196980
ANO6	2.02019	5.48E-01	0.00360	3.01E-04	0.00307	2.43E-04	0.00142	3.56E-05	QT01156603
ANO7	n.d.		n.d.		n.d.		n.d.		QT01882755
ANO8	trace		trace		trace		trace		QT00062559
ANO9	n.d.		n.d.		n.d.		n.d.		QT01841196
AQP1	trace		n.d.		0.00108	4.25E-05	trace		QT00013237
AQP10	0.03358	1.24E-02	0.00049	2.22E-05	0.00132	4.18E-05	trace		QT00043393
AQP11	n.d.		trace		trace		trace		QT00230636
AQP12A	n.d.		n.d.		n.d.		n.d.		QT00236432
AQP12B	n.d.		0.00026	3.80E-06	n.d.		trace		QT00219331
AQP2	n.d.		n.d.		trace		trace		QT00022757
AQP3	n.d.		0.00263	3.82E-04	0.00050	1.18E-05	0.00202	1.58E-04	QT00212996
AQP4	n.d.		n.d.		n.d.		n.d.		QT00054761
AQP5	n.d.		n.d.		n.d.		n.d.		QT00036099
AQP6	n.d.		n.d.		n.d.		n.d.		QT00010633

AQP7	n.d.		n.d.		n.d.		n.d.		QT01672006
AQP8	n.d.		n.d.		trace		trace		QT00039123
AQP9	n.d.		n.d.		n.d.		n.d.		QT00017710
BEST1	n.d.		trace		n.d.		n.d.		QT00023282
BEST2	n.d.		n.d.		n.d.		n.d.		QT00063889
BEST3	0.02459	1.56E-03	trace		trace		n.d.		QT01884141
BEST4	n.d.		n.d.		n.d.		n.d.		QT00029638
BSND	n.d.		n.d.		n.d.		n.d.		QT00025053
CACNA1A	n.d.		0.00020	1.92E-05	n.d.		n.d.		QT01870050
CACNA1B	n.d.		n.d.		trace		n.d.		QT00077042
CACNA1C	n.d.		n.d.		n.d.		n.d.		QT00053480
CACNA1D	n.d.		trace		n.d.		n.d.		QT00076657
CACNA1E	n.d.		n.d.		n.d.		n.d.		QT00063994
CACNA1F	n.d.		n.d.		n.d.		n.d.		QT00163093
CACNA1G	n.d.		trace		trace		trace		QT01870057
CACNA1H	n.d.		n.d.		n.d.		n.d.		QT01870064
CACNA1I	n.d.		n.d.		trace		n.d.		QT01870071
CACNA1S	n.d.		n.d.		n.d.		n.d.		QT00000833
CACNA2D1	n.d.		n.d.		n.d.		trace		QT00044968
CACNA2D2	n.d.		n.d.		n.d.		n.d.		QT00068999
CACNA2D3	n.d.		n.d.		trace		n.d.		QT00056350
CACNA2D4	n.d.		trace		trace		n.d.		QT00004592
CACNB1	trace		trace		trace		trace		QT00997808
CACNB2	n.d.		n.d.		trace		n.d.		QT00011256
CACNB3	n.d.		0.00024	6.01E-05	0.00086	1.13E-04	0.00005	3.88E-06	QT00040922
CACNB4	n.d.		n.d.		n.d.		trace		QT00047600
CACNG1	n.d.		n.d.		n.d.		n.d.		QT00028301
CACNG2	n.d.		n.d.		n.d.		n.d.		QT01014517
CACNG3	n.d.		0.00021	1.01E-05	trace		trace		QT00002919
CACNG4	n.d.		n.d.		n.d.		n.d.		QT00090048
CACNG5	n.d.		n.d.		n.d.		n.d.		QT00042245
CACNG6	n.d.		n.d.		n.d.		n.d.		QT00217441
CACNG7	n.d.		n.d.		n.d.		n.d.		QT00091315

CACNG8	n.d.		trace		trace		n.d.		QT00079009
CATSPER1	trace		0.00134	3.97E-05	0.00063	8.36E-05	0.00020	6.09E-06	QT00027965
CATSPER2	n.d.		0.00013	3.17E-06	0.00077	7.50E-06	0.00025	5.45E-05	QT00056637
CATSPER3	n.d.		0.00040	4.22E-05	0.00023	2.05E-05	0.00047	3.09E-06	QT00064099
CATSPER4	n.d.		n.d.		n.d.		n.d.		QT00020755
CCT8L2	n.d.		0.00095	5.55E-05	trace		trace		QT02309265
CFTR	n.d.		n.d.		n.d.		n.d.		QT00070007
Chrna1	n.d.		n.d.		n.d.		n.d.		QT00066017
CHRNA2	0.02330		n.d.		n.d.		n.d.		QT00010997
Chrna3	n.d.	5.93E-03	n.d.		n.d.		trace		QT00015386
Chrna4	n.d.		n.d.		n.d.		n.d.		QT00003871
Chrna5	n.d.		0.00176		0.00296		0.00360		QT00001071
Chrna6	n.d.		n.d.	2.93E-05	n.d.	1.06E-04	trace	1.22E-04	QT00017458
Chrna7	n.d.		n.d.		n.d.		n.d.		QT00074732
Chrna9	n.d.		n.d.		n.d.		trace		QT02589349
Chrna10	n.d.		n.d.		trace		trace		QT02589342
CHRNB1	n.d.		trace		trace		trace		QT00048867
CHRNB2	n.d.		n.d.		n.d.		n.d.		QT00001491
CHRNB3	n.d.		n.d.		n.d.		n.d.		QT00016170
CHRNB4	n.d.		n.d.		n.d.		n.d.		QT00010570
CHRND	n.d.		n.d.		n.d.		n.d.		QT00027412
CHRNE	n.d.		0.00047	6.20E-05	0.00036	4.72E-05	trace		QT00049644
CHRNG	n.d.		n.d.		n.d.		n.d.		QT00034587
CLCA1	n.d.		n.d.		n.d.		0.04487	1.39E-03	QT01666889
CLCA2	n.d.		n.d.		trace		0.00022	1.76E-05	QT00029155
CLCA3	n.d.		n.d.		n.d.		0.00063	2.40E-05	QT00197736
CLCA4	n.d.		n.d.		n.d.		n.d.		QT00090727
CLCC1	n.d.		0.00476	1.81E-04	0.00363	1.29E-04	0.00940	4.80E-05	QT00015827
CLCN1	n.d.		n.d.		n.d.		n.d.		QT00020314
CLCN2	n.d.		0.00005	8.05E-06	0.00029	3.79E-05	0.00020	1.10E-06	QT00055804
CLCN3	0.58124	1.39E-01	0.00416	2.03E-04	0.00303	1.56E-04	0.00148	1.01E-04	QT00079289
CLCN4	0.03249	2.56E-03	0.00482	6.24E-04	0.00092	1.14E-04	trace		QT00071008
CLCN5	n.d.		trace		0.00026	5.95E-06	0.00046	1.17E-05	QT00998683

CLCN6	trace		trace		0.00048	9.30E-05	0.00034	2.85E-05	QT00039277
CLCN7	trace		0.00979	5.03E-04	0.00445	4.41E-04	0.00450	2.32E-04	QT00076307
CLCNKA	n.d.		n.d.		trace		n.d.		QT01679258
CLCNKB	n.d.		n.d.		n.d.		n.d.		QT01666854
CLIC1	0.24101	3.55E-02	0.23383	1.78E-02	0.06624	3.40E-03	0.08270	5.92E-04	QT01848833
CLIC2	n.d.		0.00045	7.43E-05	0.01550	4.87E-04	0.04991	5.61E-04	QT00998711
CLIC3	trace		n.d.		n.d.		n.d.		QT00011781
CLIC4	2.75848	4.81E-01	0.06065	3.79E-03	0.09624	3.47E-03	0.05026	2.29E-03	QT02312569
CLIC5	n.d.		n.d.		n.d.		trace		QT00070910
CLIC6	n.d.		n.d.		n.d.		trace		QT00077826
CLNS1A	n.d.		0.10656	6.11E-04	0.06830	1.55E-03	0.07499	8.96E-04	QT00068145
CNGA1	n.d.		n.d.		n.d.		trace		QT00000875
CNGA2	n.d.		trace		n.d.		n.d.		QT00067522
CNGA3	n.d.		n.d.		n.d.		n.d.		QT00028245
CNGA4	n.d.		trace		n.d.		n.d.		QT02290519
CNGB1	n.d.		trace		n.d.		n.d.		QT00024605
CNGB3	n.d.		n.d.		n.d.		n.d.		QT00027349
FXYD1	0.02500	8.52E-03	trace		trace		trace		QT00052038
FXYD2	n.d.		trace		n.d.		trace		QT00057841
FXYD3	n.d.		n.d.		n.d.		n.d.		QT00040061
FXYD4	n.d.		n.d.		n.d.		n.d.		QT01021055
FXYD5	0.34529	9.22E-02	0.04246	9.52E-04	0.06749	5.09E-03	0.02944	8.31E-04	QT00041489
FXYD6	n.d.		n.d.		n.d.		trace		QT00088186
FXYD7	trace		0.00116	9.35E-05	trace		trace		QT01021041
GABRA1	n.d.		n.d.		n.d.		n.d.		QT00030002
GABRA2	n.d.		0.00032	6.11E-05	trace		trace		QT00019376
GABRA3	n.d.		trace		trace		n.d.		QT00020636
GABRA4	n.d.		n.d.		n.d.		n.d.		QT00032046
GABRA5	n.d.		n.d.		n.d.		n.d.		QT01670109
GABRA6	n.d.		n.d.		trace		trace		QT00015596
GABRB1	n.d.		n.d.		n.d.		n.d.		QT00007455
GABRB2	n.d.		trace		trace		trace		QT00015050
GABRB3	n.d.		n.d.		n.d.		0.00059	1.38E-05	QT00036155

GABRD	trace		n.d.		n.d.		n.d.		QT00019005
GABRE	n.d.		0.00086	5.08E-05	0.00031	4.96E-05	trace		QT00006349
GABRG1	n.d.		n.d.		n.d.		n.d.		QT00058765
GABRG2	n.d.		n.d.		n.d.		n.d.		QT00002905
GABRG3	n.d.		n.d.		n.d.		n.d.		QT01001014
GABRP	n.d.		n.d.		n.d.		n.d.		QT00007182
GABRQ	n.d.		n.d.		trace		trace		QT00038472
GABRR1	n.d.		n.d.		n.d.		n.d.		QT00059444
GABRR2	trace		n.d.		0.00037	1.38E-05	trace		QT00017038
GABRR3	n.d.		n.d.		n.d.		n.d.		QT00243215
GJA1	n.d.		0.00024	6.94E-05	0.00040	6.92E-06	trace		QT00012684
GJA10	n.d.		trace		n.d.		trace		QT01027817
GJA3	n.d.		trace		n.d.		trace		QT01001301
GJA4	trace		0.01233	2.65E-03	0.00134	1.19E-04	trace		QT00212093
GJA5	n.d.		0.00042	2.65E-03	0.00152	1.17E-04	trace		QT01001308
GJA8	n.d.		0.00069	6.31E-05	trace		trace		QT02306493
GJA9	n.d.		0.00084	1.39E-04	trace		trace		QT01026543
GJB1	n.d.		0.00038	6.27E-05	n.d.		n.d.		QT00213255
GJB2	n.d.		0.00011	1.50E-05	0.00042	2.46E-05	trace		QT00244531
GJB3	n.d.		0.00036	6.77E-05	trace		n.d.		QT00241108
GJB4	trace		n.d.		0.00031	9.16E-06	trace		QT01030414
GJB5	trace		0.00771	4.01E-04	0.00361	1.06E-04	n.d.		QT01001343
GJB6	n.d.		n.d.		n.d.		n.d.		QT00072387
GJB7	n.d.		n.d.		n.d.		n.d.		QT00024577
GJC1	n.d.		0.00085	4.04E-05	trace		0.00020	1.82E-05	QT00239659
GJC2	n.d.		n.d.		n.d.		n.d.		QT01674239
GJC3	n.d.		0.00021	2.39E-05	0.00033	3.62E-05	trace		QT00046795
GJD2	n.d.		n.d.		n.d.		trace		QT00493115
GJD3	n.d.		0.00023	4.16E-05	trace		n.d.		QT00219415
GJD4	n.d.		trace		n.d.		n.d.		QT00081032
GLRA1	n.d.		n.d.		n.d.		n.d.		QT00020335
GLRA2	trace		n.d.		n.d.		n.d.		QT00001645
GLRA3	n.d.		n.d.		n.d.		trace		QT00056749

GLRA4	trace		n.d.		n.d.		n.d.		QT00097706
GLRB	trace		0.00085	4.91E-05	0.00330	3.04E-04	0.00050	4.29E-05	QT00008337
GPM6A	n.d.		n.d.		n.d.		n.d.		QT00013139
GRIA1	n.d.		n.d.		n.d.		n.d.		QT01001665
GRIA2	n.d.		n.d.		n.d.		n.d.		QT00008274
GRIA3	n.d.		n.d.		n.d.		n.d.		QT00050092
GRIA4	n.d.		n.d.		n.d.		n.d.		QT00072240
GRID1	n.d.		n.d.		trace		n.d.		QT00093023
GRID2	n.d.		n.d.		n.d.		n.d.		QT00084462
GRIK1	n.d.		n.d.		trace		trace		QT00080332
GRIK2	n.d.		n.d.		n.d.		n.d.		QT00016597
GRIK3	n.d.		n.d.		n.d.		n.d.		QT00001526
GRIK4	trace		trace		0.00091	1.07E-04	n.d.		QT00099897
GRIK5	trace		0.00128	6.60E-06	0.00082	7.26E-06	0.00104	5.76E-05	QT00052339
GRIN1	n.d.		n.d.		n.d.		n.d.		QT00082089
GRIN2A	n.d.		n.d.		n.d.		n.d.		QT00050379
GRIN2B	n.d.		n.d.		n.d.		n.d.		QT00059619
GRIN2C	n.d.		trace		n.d.		n.d.		QT00051905
GRIN2D	trace		0.00023	2.68E-05	trace		trace		QT00065324
GRIN3A	n.d.		n.d.		n.d.		n.d.		QT00043617
GRIN3B	n.d.		n.d.		n.d.		trace		QT00055972
GRINA	trace		0.00779	4.80E-04	0.02762	1.15E-03	0.02539	1.73E-04	QT00217490
HCN1	n.d.		n.d.		n.d.		trace		QT00048020
HCN2	n.d.		trace		n.d.		trace		QT01668723
HCN3	n.d.		0.00122	9.99E-06	0.00078	5.30E-05	0.00013	2.33E-05	QT01672958
HCN4	n.d.		n.d.		n.d.		n.d.		QT00038108
HTR3A	n.d.		n.d.		n.d.		n.d.		QT00061397
HTR3B	n.d.		n.d.		n.d.		n.d.		QT00034538
HTR3C	n.d.		n.d.		n.d.		n.d.		QT00053543
HTR3D	n.d.		0.00114	1.84E-04	trace		trace		QT01154118
HTR3E	n.d.		n.d.		n.d.		n.d.		QT00075047
HVCN1	trace		0.00052	9.44E-05	0.00024	3.98E-05	trace		QT01027558
ITPR1	0.01537	1.46E-03	0.00192	5.37E-04	0.00105	5.43E-05	0.00045	1.79E-05	QT00056490

ITPR2	0.08799	3.44E-02	0.00020	3.17E-05	0.00049	3.24E-05	0.00025	3.00E-05	QT01336468
ITPR3	n.d.		n.d.		n.d.		trace		QT00011865
KCNA1	n.d.		0.00040	1.99E-05	n.d.		n.d.		QT01195733
KCNA10	n.d.		0.00054	7.75E-05	n.d.		trace		QT00209279
KCNA2	trace		n.d.		n.d.		trace		QT00035707
KCNA3	0.24943	5.35E-02	n.d.		n.d.		trace		QT01667918
KCNA4	n.d.		0.00021	4.23E-05	n.d.		n.d.		QT00224112
KCNA5	n.d.		0.00057	4.80E-05	trace		trace		QT01003177
KCNA6	n.d.		trace		n.d.		n.d.		QT00215565
KCNA7	n.d.		0.00046	8.82E-05	trace		n.d.		QT01003184
KCNAB1	trace		trace		0.00033	5.03E-05	n.d.		QT00031136
KCNAB2	trace		0.00311	2.61E-04	0.00574	2.27E-04	0.00015	2.31E-05	QT00051961
KCNAB3	trace		trace		0.00026	3.58E-05	trace		QT00056931
KCNB1	n.d.		n.d.		n.d.		n.d.		QT00014749
KCNB2	n.d.		n.d.		n.d.		n.d.		QT00002688
KCNC1	n.d.		0.00043	7.22E-05	trace		trace		QT00213780
KCNC2	n.d.		n.d.		n.d.		n.d.		QT00007427
KCNC3	n.d.		n.d.		n.d.		n.d.		QT00074081
KCNC4	n.d.		trace		0.00021	8.26E-06	trace		QT01669150
KCND1	n.d.		0.00049	3.73E-05	0.00039	4.35E-05	0.00017	1.05E-05	QT00010773
KCND2	n.d.		n.d.		n.d.		n.d.		QT01870414
KCND3	n.d.		n.d.		n.d.		n.d.		QT00050400
KCNE1	n.d.		n.d.		n.d.		n.d.		QT01003226
KCNE1L	n.d.		0.00073	3.20E-05	0.00145	3.93E-04	trace		QT00236194
KCNE2	trace		0.00050	1.22E-06	0.00019	2.85E-05	trace		QT00100044
KCNE3	0.09149	1.65E-02	0.00568	3.10E-04	0.01053	2.92E-04	0.00081	7.73E-05	QT00093870
KCNE4	n.d.		0.00024	2.39E-05	n.d.		trace		QT00046676
KCNF1	n.d.		n.d.		n.d.		n.d.		QT00201243
KCNG1	n.d.		n.d.		n.d.		n.d.		QT00205324
KCNG2	n.d.		trace		trace		trace		QT01018430
KCNG3	n.d.		0.00073	8.78E-05	n.d.		trace		QT01032395
KCNG4	trace		n.d.		n.d.		n.d.		QT00053487
KCNH1	n.d.		trace		n.d.		n.d.		QT00092127

KCNH2	n.d.		0.00083	2.72E-05	0.00309	1.65E-04	0.00435	1.17E-04	QT01003254
KCNH3	n.d.		n.d.		n.d.		n.d.		QT00011753
KCNH4	n.d.		n.d.		trace		trace		QT00042497
KCNH5	n.d.		n.d.		n.d.		n.d.		QT00007406
KCNH6	n.d.		n.d.		n.d.		n.d.		QT00065072
KCNH7	n.d.		n.d.		n.d.		n.d.		QT00080283
KCNH8	n.d.		n.d.		n.d.		n.d.		QT00066647
KCNIP1	n.d.		n.d.		trace		n.d.		QT00078050
KCNIP2	n.d.		n.d.		n.d.		n.d.		QT00016254
KCNIP3	n.d.		n.d.		n.d.		trace		QT00060564
KCNIP4	n.d.		n.d.		n.d.		n.d.		QT01026298
KCNJ1	n.d.		trace		n.d.		n.d.		QT01003275
KCNJ10	n.d.		n.d.		n.d.		n.d.		QT00059031
KCNJ11	n.d.		0.00021	6.27E-05	0.00077	4.18E-05	0.00023	1.08E-05	QT00216475
KCNJ12	n.d.		n.d.		n.d.		n.d.		QT01668317
KCNJ13	n.d.		n.d.		n.d.		n.d.		QT00229257
KCNJ14	n.d.		trace		n.d.		n.d.		QT00207452
KCNJ15	n.d.		0.00249	2.88E-04	0.00056	6.90E-05	0.00004	4.36E-06	QT00062447
KCNJ16	n.d.		n.d.		n.d.		trace		QT00071687
KCNJ2	n.d.		n.d.		n.d.		n.d.		QT00001022
KCNJ3	n.d.		n.d.		trace		n.d.		QT00030380
KCNJ4	n.d.		0.00115	6.78E-05	0.00040	2.60E-05	n.d.		QT00201845
KCNJ5	n.d.		trace		n.d.		n.d.		QT00070406
KCNJ6	n.d.		trace		n.d.		n.d.		QT00010444
KCNJ8	n.d.		0.00055	1.02E-04	0.00122	4.42E-05	0.00060	5.87E-05	QT01157996
KCNJ9	n.d.		n.d.		n.d.		n.d.		QT00011935
KCNK1	trace		n.d.		0.00014	2.19E-05	0.00813	1.22E-04	QT00039396
KCNK10	n.d.		n.d.		n.d.		n.d.		QT00038899
KCNK12	n.d.		n.d.		n.d.		n.d.		QT00209258
KCNK13	n.d.		0.00046	9.16E-05	trace		trace		QT00232708
KCNK15	n.d.		n.d.		n.d.		n.d.		QT00238658
KCNK16	n.d.		n.d.		n.d.		n.d.		QT00043666
KCNK17	trace		0.01814	1.46E-03	0.00408	7.40E-04	trace		QT00036470

KCNK18	n.d.		0.00083	7.49E-05	trace		trace		QT01034348
KCNK2	n.d.		n.d.		n.d.		n.d.		QT00080710
KCNK3	n.d.		trace		n.d.		n.d.		QT00201264
KCNK4	n.d.		trace		n.d.		n.d.		QT01670151
KCNK5	n.d.		0.00293	3.47E-04	0.00323	9.50E-05	0.00124	2.86E-05	QT00069188
KCNK6	0.03477	8.35E-03	0.00149	2.06E-04	0.00263	8.56E-05	0.00189	2.62E-05	QT00210791
KCNK7	n.d.		n.d.		n.d.		trace		QT01674127
KCNK9	n.d.		0.00057	1.13E-04	trace		trace		QT01020369
KCNMA1	trace		n.d.		trace		n.d.		QT00024157
KCNMB1	0.02491	9.26E-03	0.00061	3.31E-05	0.00077	1.02E-05	n.d.		QT00080493
KCNMB2	n.d.		n.d.		trace		n.d.		QT00083097
KCNMB3	trace		0.00201	1.29E-04	0.00048	1.95E-05	0.00042	4.62E-06	QT00094276
KCNMB4	0.00925	2.78E-03	0.00081	1.21E-04	0.00141	1.05E-04	0.00010	1.78E-05	QT00097496
KCNN1	n.d.		0.00093	5.36E-05	0.00028	3.33E-05	trace		QT00025375
KCNN2	n.d.		trace		trace		trace		QT00016611
KCNN3	trace		trace		trace		n.d.		QT00070966
KCNN4	n.d.		0.00107	1.43E-05	0.00474	6.64E-04	0.01038	1.60E-04	QT00003780
KCNQ1	n.d.		0.00031	3.48E-05	n.d.		n.d.		QT00016065
KCNQ2	n.d.		n.d.		n.d.		n.d.		QT00036463
KCNQ3	n.d.		n.d.		n.d.		n.d.		QT00053865
KCNQ4	trace		n.d.		0.00028	7.87E-05	0.00028	1.60E-05	QT00073066
KCNQ5	n.d.		n.d.		0.00198	1.44E-04	0.00205	3.05E-05	QT00028280
KCNRG	trace		n.d.		trace		n.d.		QT00229999
KCNS1	n.d.		n.d.		n.d.		n.d.		QT00019621
KCNS2	n.d.		trace		n.d.		trace		QT00219170
KCNS3	n.d.		trace		trace		trace		QT00061411
KCNT1	n.d.		n.d.		n.d.		n.d.		QT00017801
KCNT2	trace		n.d.		trace		n.d.		QT00063707
KCNU1	n.d.		n.d.		n.d.		n.d.		QT01666651
KCNV1	n.d.		n.d.		trace		0.00022	2.86E-05	QT00004487
KCNV2	n.d.		0.00169	3.59E-05	0.00066	6.70E-05	n.d.		QT01032304
KCTD1	n.d.		n.d.		trace		n.d.		QT00096565
KCTD10	0.03547	9.84E-03	0.00056	5.51E-05	0.00137	4.28E-05	0.00111	2.93E-05	QT00088746

KCTD11	trace		0.00004	2.04E-06	0.00001	1.22E-06	0.00011	2.01E-05	QT01031394
KCTD12	n.d.		0.00298	1.47E-04	0.00031	6.04E-05	0.00191	5.87E-05	QT00204771
KCTD13	0.01560	3.88E-03	0.00287	2.74E-04	0.00448	2.28E-04	0.00321	3.35E-04	QT01033270
KCTD14	n.d.		0.00045	1.04E-04	0.00019	1.21E-05	0.00043	6.80E-05	QT00200872
KCTD15	n.d.		0.00073	1.00E-04	0.02099	3.79E-04	0.01156	3.46E-04	QT00028168
KCTD16	n.d.		0.00079	1.63E-04	trace		0.00006	7.31E-06	QT00231560
KCTD17	n.d.		n.d.		n.d.		trace		QT00091798
KCTD18	0.02210	2.86E-03	0.00151	7.49E-05	0.00180	5.91E-05	0.00086	1.70E-04	QT00048251
KCTD19	n.d.		n.d.		trace		trace		QT01152921
KCTD2	0.01617	2.93E-03	0.00095	1.83E-04	0.00148	7.49E-05	0.00144	5.17E-05	QT00071470
KCTD20	0.05130	4.07E-03	0.00047	4.10E-05	0.00080	8.45E-05	0.00119	3.53E-05	QT00046928
KCTD21	n.d.		0.00142	5.90E-05	0.00114	1.62E-04	0.00142	3.83E-05	QT00235060
KCTD3	n.d.		0.00128	1.14E-04	0.00198	1.12E-04	0.00081	2.89E-05	QT00041517
KCTD4	n.d.		0.00055	5.43E-05	0.00015	2.34E-05	n.d.		QT00201222
KCTD5	trace		0.00153	9.42E-05	0.00167	5.41E-05	0.00157	1.05E-04	QT00011564
KCTD6	n.d.		0.00050	4.98E-05	0.00059	7.98E-06	0.00057	4.87E-05	QT00200746
KCTD7	trace		n.d.		trace		trace		QT00085267
KCTD8	n.d.		n.d.		n.d.		n.d.		QT00084441
KCTD9	0.00441	2.65E-04	0.00250	6.35E-05	0.00363	1.47E-04	0.00207	1.98E-04	QT01670046
LRRC8A	n.d.		0.00069	8.79E-05	0.00069	5.66E-06	0.00083	1.97E-04	QT01023302
LRRC8B	0.20202	7.60E-02	0.00266	2.42E-04	0.00328	1.81E-04	0.00432	8.27E-04	QT01017450
LRRC8C	n.d.		0.00028	3.62E-05	0.00052	1.88E-05	0.00162	6.36E-05	QT00055720
LRRC8D	n.d.		0.00187	7.10E-05	0.00224	9.67E-05	0.00280	6.54E-05	QT01679202
LRRC8E	n.d.		n.d.		trace		trace		QT00079639
MCOLN1	n.d.		0.00177	7.61E-05	0.00330	1.05E-04	0.00228	4.51E-05	QT00094234
MCOLN2	n.d.		trace		0.00337	4.66E-04	trace		QT00072849
MCOLN3	n.d.		n.d.		n.d.		n.d.		QT00100835
NALCN	n.d.		0.00022	4.52E-05	trace		n.d.		QT00096537
ORAI1	0.12405	2.45E-02	0.00435	9.17E-04	0.00289	3.20E-04	0.00142	3.10E-05	QT00202587
ORAI2	trace		trace		trace		trace		QT00215229
ORAI3	n.d.		0.00671	2.57E-04	0.00333	1.66E-05	0.00092	9.60E-06	QT00231910
P2RX1	0.39701	1.75E-01	0.00659	2.82E-04	0.00232	2.52E-04	0.00069	2.95E-05	QT00009240
P2RX2	n.d.		n.d.		n.d.		n.d.		QT00493682

P2RX3	n.d.		n.d.		n.d.		n.d.		QT00026446
P2RX4	trace		0.00778	1.48E-03	0.00136	5.10E-05	0.00289	4.28E-05	QT00049693
P2RX5	n.d.		n.d.		0.00170	9.92E-05	0.00128	1.20E-04	QT00057785
P2RX6	trace		trace		0.00005	1.28E-05	n.d.		QT00018949
P2RX7	n.d.		0.00088	7.24E-05	0.00053	2.99E-05	0.00060	1.25E-04	QT00083643
PANX1	0.11326	3.34E-02	trace		0.00017	5.19E-06	trace		QT00085925
PANX2	n.d.		0.00040	7.35E-05	trace		n.d.		QT00219359
PANX3	n.d.		n.d.		trace		n.d.		QT00016884
PIEZO1	trace		0.00505	1.16E-04	0.01099	2.44E-03	0.00884	8.79E-05	QT00088403
PIEZO2	n.d.		n.d.		0.00150	3.10E-04	0.00687	2.59E-04	QT02359623
PKD1	trace		0.02618	4.94E-04	0.00675	1.23E-03	0.00732	6.07E-05	QT00013132
PKD1L1	n.d.		n.d.		trace		n.d.		QT00044429
PKD1L2	n.d.		n.d.		n.d.		n.d.		QT00059094
PKD1L3	n.d.		trace		n.d.		n.d.		QT00067662
PKD2	n.d.		0.00161	2.37E-05	0.00248	1.84E-04	0.00100	2.56E-05	QT00027853
PKD2L1	n.d.		n.d.		n.d.		n.d.		QT00037667
PKD2L2	n.d.		n.d.		n.d.		n.d.		QT00038675
PKDREJ	n.d.		n.d.		n.d.		trace		QT00215901
RYR1	n.d.		trace		0.00019	3.03E-05	n.d.		QT00079002
RYR2	n.d.		n.d.		trace		0.00016	1.79E-05	QT00018368
RYR3	n.d.		n.d.		trace		trace		QT00092456
SCN10A	n.d.		n.d.		n.d.		n.d.		QT01008028
SCN11A	n.d.		n.d.		trace		n.d.		QT01016344
SCN1A	n.d.		n.d.		n.d.		n.d.		QT02308355
SCN1B	trace		n.d.		trace		n.d.		QT00066080
SCN2A	n.d.		trace		trace		trace		QT00070707
SCN2B	n.d.		0.00049	9.14E-05	trace		trace		QT01667687
SCN3A	n.d.		n.d.		trace		n.d.		QT01671264
SCN3B	trace		n.d.		n.d.		n.d.		QT00002184
SCN4A	n.d.		n.d.		n.d.		0.00011	5.65E-06	QT00009765
SCN4B	n.d.		n.d.		n.d.		n.d.		QT00011802
SCN5A	trace		n.d.		0.00010	2.57E-06	trace		QT00091812
SCN7A	n.d.		n.d.		n.d.		n.d.		QT00034132

SCN8A	n.d.		n.d.		n.d.		n.d.		QT00020923
SCN9A	n.d.		0.00015	3.28E-05	0.00195	6.23E-04	0.00034	2.61E-05	QT00001505
SCNN1A	trace		n.d.		trace		n.d.		QT00022883
SCNN1B	n.d.		n.d.		n.d.		n.d.		QT00051597
SCNN1D	n.d.		n.d.		n.d.		n.d.		QT00086051
SCNN1G	n.d.		n.d.		n.d.		n.d.		QT00063217
STIM1	0.08587	1.96E-02	0.00877	1.06E-03	0.00310	3.71E-04	0.00080	1.96E-05	QT00083538
STIM2	trace		0.00539	2.71E-04	0.00294	1.53E-04	0.00227	7.55E-05	QT00023744
STX1B	trace		n.d.		n.d.		trace		QT00058793
TMEM109	0.00677	6.33E-04	0.00187	6.36E-05	0.00358	2.47E-04	0.00396	6.33E-05	QT00004571
TMEM37	n.d.		0.00029	3.38E-05	trace		trace		QT01530361
TMEM38A	n.d.		trace		n.d.		n.d.		QT00063931
TMEM38B	n.d.		0.01118	5.71E-05	0.00867	8.50E-04	0.01184	1.84E-04	QT00054208
TNFAIP1	n.d.		n.d.		n.d.		trace		QT00013216
TOMM40	n.d.		0.03045	1.25E-03	0.03004	1.65E-03	0.05683	3.82E-04	QT01674099
TPCN1	trace		0.00290	2.38E-05	0.00157	7.29E-05	0.00182	4.57E-05	QT00088760
TPCN2	n.d.		0.00037	2.88E-05	0.00070	5.77E-06	0.00030	2.65E-05	QT00084763
TRPA1	n.d.		n.d.		n.d.		n.d.		QT00026054
TRPC1	n.d.		0.00012	1.04E-05	0.00367	7.82E-05	0.00011	2.04E-06	QT01009771
TRPC3	n.d.		0.00050	8.99E-06	trace		0.00010	2.23E-06	QT00025025
TRPC4	n.d.		n.d.		n.d.		n.d.		QT00046081
TRPC5	n.d.		n.d.		n.d.		n.d.		QT00019488
TRPC6	0.05003	2.47E-02	0.00279	4.85E-04	n.d.		n.d.		QT00037660
TRPC7	n.d.		trace		n.d.		n.d.		QT00030898
TRPM1	n.d.		n.d.		n.d.		n.d.		QT00031087
TRPM2	n.d.		0.00041	5.83E-05	trace		trace		QT01870407
TRPM3	n.d.		n.d.		trace		n.d.		QT00009940
TRPM4	n.d.		0.00054	4.21E-05	0.00028	4.29E-05	trace		QT00069545
TRPM5	n.d.		n.d.		n.d.		n.d.		QT00034734
TRPM6	n.d.		n.d.		trace		0.00083	4.75E-05	QT00043456
TRPM7	n.d.		0.00137	8.92E-05	0.00207	3.93E-04	0.00043	2.91E-05	QT00082425
TRPM8	n.d.		n.d.		n.d.		n.d.		QT00038906
TRPV1	n.d.		0.00062	2.67E-05	0.00035	2.90E-05	trace		QT00046109

TRPV2	n.d.		0.00456	6.80E-05	0.00884	4.09E-04	0.00533	4.39E-05	QT00035987
TRPV3	n.d.		0.00115	1.92E-04	trace		trace		QT00070448
TRPV4	n.d.		n.d.		n.d.		n.d.		QT00077217
TRPV5	n.d.		n.d.		n.d.		trace		QT00029442
TRPV6	n.d.		n.d.		n.d.		n.d.		QT00040096
TTYH1	n.d.		n.d.		trace		n.d.		QT00035882
TTYH2	n.d.		n.d.		n.d.		n.d.		QT00007504
TTYH3	n.d.		n.d.		n.d.		n.d.		QT00058191
VDAC1	n.d.		0.00214	6.05E-04	0.00218	4.79E-05	0.02448	9.79E-03	QT00092680
VDAC2	0.05271	1.35E-02	0.09444	1.23E-02	0.06112	2.36E-04	0.03147	3.69E-04	QT01670949
VDAC3	0.64516	2.04E-01	0.04650	4.28E-03	0.04116	8.82E-04	0.08594	1.51E-03	QT01678341
ZACN	n.d.		n.d.		n.d.		n.d.		QT01678950
GAPDH	NA		NA		NA		NA		QT01192646
PTPRC [CD45]	NA		NA		NA		NA		QT01869931
GYPA	NA		NA		NA		NA		QT00053109
GPIBB	NA		NA		NA		NA		QT01001441

Values represent expression levels relative to GAPDH, calculated as described in the methods section,
for platelets (PLT), MEG-01, CHRF-288-11 (CHRF) and HEL cells.
n.d., not detected; trace, gene detected at a level above background but insufficient for quantification.
Values represent the mean +/- SEM from 4 donors (platelets) or 3 separate batches of each cell line.
The QuantiTect assay identification number (Qiagen) for each gene is shown in the far right column.
